# Generation of a Spiral Ganglion Neuron Degeneration Mouse Model

**DOI:** 10.3389/fcell.2021.761847

**Published:** 2021-10-27

**Authors:** Zhengqing Hu, Fnu Komal, Aditi Singh, Meng Deng

**Affiliations:** ^1^John D. Dingell VA Medical Center, Detroit, MI, United States; ^2^Department of Otolaryngology-HNS, Wayne State University School of Medicine, Detroit, MI, United States

**Keywords:** auditory brainstem response, degeneration, iDTR, neurofilament, spiral ganglion, Cre-LoxP

## Abstract

Spiral ganglion neurons (SGNs) can be injured by a wide variety of insults. However, there still is a lack of degeneration models to specifically damage the SGNs without disturbing other types of cells in the inner ear. This study aims to generate an SGN-specific damage model using the Cre-LoxP transgenic mouse strains. The Cre-inducible diphtheria toxin receptor (*iDTR^+/+^*) knock-in mouse strain was crossed with a mouse strain with Cre activity specific to neurons (*Nefl*^*CreER/CreER*^). Expression of the Cre-recombinase activity was evaluated using the reporter mouse strain Ai9 at pre-hearing, hearing onset, and post-hearing stages. Accordingly, heterozygous *Nefl*^*CreER/*+^;iDTR^+/–^ mice were treated with tamoxifen on postnatal days 1–5 (P1–5), followed by diphtheria toxin (DT) or vehicle injection on P7, P14, and P21 to evaluate the SGN loss. Robust tamoxifen-induced Cre-mediated Ai9 tdTomato fluorescence was observed in the SGN area of heterozygous *Nefl*^*CreER/*+^;Ai9^+/–^ mice treated with tamoxifen, whereas vehicle-treated heterozygote mice did not show tdTomato fluorescence. Compared to vehicle-treated *Nefl*^*CreER/*+^;iDTR^+/–^ mice, DT-treated *Nefl*^*CreER/*+^;iDTR^+/–^ mice showed significant auditory brainstem response (ABR) threshold shifts and SGN cell loss. Hair cell count and functional study did not show significant changes. These results demonstrate that the *Nefl*^*CreER/CreER*^ mouse strain exhibits inducible SGN-specific Cre activity in the inner ear, which may serve as a valuable SGN damage model for regeneration research of the inner ear.

## Introduction

In the auditory system, spiral ganglion neurons (SGNs) are bipolar neurons that transfer auditory signals from auditory hair cells to the cochlear nucleus in the brainstem (Echteler, 1992; Nayagam et al., 2011). SGNs are sensitive to a variety of insults, including sound overstimulation, genetic disorders, aging, ototoxic drugs, and trauma (Ylikoski et al., 1998; Carignano et al., 2019; Wu et al., 2021). Degeneration of SGNs usually causes irreversible sensorineural hearing loss, in which the auditory signals perceived by hair cells are not able to transfer to the cochlear nucleus. It is essential to establish an SGN damage model to understand the degeneration of SGNs. This would provide fundamental knowledge to guide the prevention of SGN damage and the regeneration of SGNs to conduct auditory signals from the inner ear to the brainstem. Currently, knowledge on the SGN degeneration model is very limited.

SGNs receive auditory signals from hair cells; therefore, injuries to hair cells often cause secondary damage to SGNs (Johnsson, 1974; Pan et al., 2017). For instance, ototoxic drugs, including aminoglycoside and cisplatin, cause hair cell damage, which leads to secondary damage to SGNs (Dallos and Harris, 1978; Breglio et al., 2017). In other circumstances, aging can cause progressive hair cell degeneration, which subsequently injures SGNs as a secondary degeneration (Carignano et al., 2019; Wu et al., 2021). Additionally, some ototoxic drugs (e.g., neomycin), aging, and other insults can directly damage SGNs (Majumder et al., 2017; Zhong et al., 2020). The combination of primary and secondary patterns complicates the mechanisms of SGN degeneration. Therefore, it is necessary to develop an approach only targeting SGNs without interfering with hair cells.

The Cre-LoxP system provides the opportunity to target cell types expressing a tissue-specific gene (Nakamura et al., 2006; Rotheneichner et al., 2017; Jahn et al., 2018). In a previous study, *Bhlhb5*^*Cre*/+^ mice that showed Cre activity in SGNs were bred with mice expressing the Cre-inducible diphtheria toxin receptor (iDTR; iDTR^+/+^ mice) (Pan et al., 2017). It was found that diphtheria toxin (DT) injection caused 30–40% SGN damage in *Bhlhb5*^*Cre*/+^;iDTR^+/–^ offspring. It is known that SGN development continues during the postnatal period up to postnatal day 28 (P28) (Shrestha et al., 2018; Sun et al., 2018). However, in the aforementioned study, DT was injected on P21. It remains unclear whether an early postnatal or pre-hearing DT injection would damage SGNs and whether SGN damage is consistent or recovered during postnatal development. Moreover, significant auditory brainstem response (ABR) threshold changes were not observed in DT-treated *Bhlhb5*^*Cre/*+^;iDTR^+/–^ offspring. Therefore, the generation of an SGN loss model with significant functional ABR threshold shifts remains a challenge.

In this study, a mouse strain with the estrogen receptor tamoxifen 2-inducible Cre cassette knocked into the *Nefl* gene (*Nefl*^*CreER/CreER*^) was bred with the iDTR mouse strain. *Nefl* encodes neurofilament light chain (Nefl), which is a major neuronal cytoskeleton component expressed in the soma, dendrites, and axon of developing and mature neurons, including the neurons along the auditory pathway (Liem, 1993; Illing, 2001; Liu et al., 2004). In the cochlea, *Nefl* is expressed in SGNs, but not in other types of cells such as hair cells (Torkos et al., 2008; Sun et al., 2018). When bred with mice expressing the Cre-inducible iDTR, the SGNs of *Nefl*^*Cre/*+^;iDTR^+/–^ offspring were expected to be specifically damaged following DT treatment without interfering with hair cells. To determine whether SGN damage occurred before, around, or after hearing onset, DT was administered on postnatal days 7, 14, and 21, respectively. Functional, morphological, and protein expression assays were used to evaluate SGN damage following DT treatment.

## Materials and Methods

### Animals and Genotyping

The experimental procedures on animals were approved by the Institutional Animal Care and Use Committee (IACUC) at Wayne State University. The *Nefl*^*CreER*/CreER^ (stock no. 008363), iDTR (stock no. 007900), and the reporter Ai9 (stock no. 007909) mouse strains were obtained from Jackson Laboratories (Bar Harbor, ME, United States) (Buch et al., 2005; Rotolo et al., 2008; Madisen et al., 2010). They were maintained and bred following the guidelines of the local Division of Laboratory Animal Resources. *Nefl*^*CreER/CreER*^ mice were crossed with iDTR or Ai9 mice, followed by genotyping. Heterozygous *Nefl*^*CreER/*+^;iDTR^+/–^ and *Nefl*^*CreER/*+^;Ai9^+/–^ mice were used in this study. The homozygous animals were used for breeding and maintenance of the strains.

A tail snip-based genotyping was performed to determine the genotypes of the mice (Fang et al., 2012). Two millimeters of the tail was snipped and placed in alkaline lysis buffer (25 mM NaOH and 0.2 mM ethylenediaminetetraacetic acid (EDTA; E5134, Sigma, St. Louis, MO, United States) for the hotshot procedure of 98°C for 1 h, followed by neutralization (40 mM Tris-HCl; Sigma) for 5 min at room temperature to harvest gDNA in the supernatant. Allele-specific PCR was used to determine the genotypes of the mice using the vendor’s protocols. The primers included: *Nefl*^*CreER/CreER*^: common, ATT ATT ATT GTA AAC ATC TGT GTG ATT CA; mutant forward, CGC ATA GAA ATT GCA TCA ACG CAT; and wild type reverse, AGA GGA GCA GGT GGC TAA GAA GAA AGA; Ai9: wild type forward, AAG GGA GCT GCA GTG GAG TA; wild type reverse, CCG AAA ATC TGT GGG AAG TC; mutant forward, CTG TTC CTG TAC GGC ATG G; and mutant reverse, GGC ATT AAA GCA GCG TAT CC; iDTR: common, AAA GTC GCT CTG AGT TGT TAT; mutant, GCG AAG AGT TTG TCC TCA ACC; and wild type reverse, GGA GCG GGA GAA ATG GAT ATG.

### Tamoxifen and Diphtheria Toxin Treatment

**FIGURE 1:**
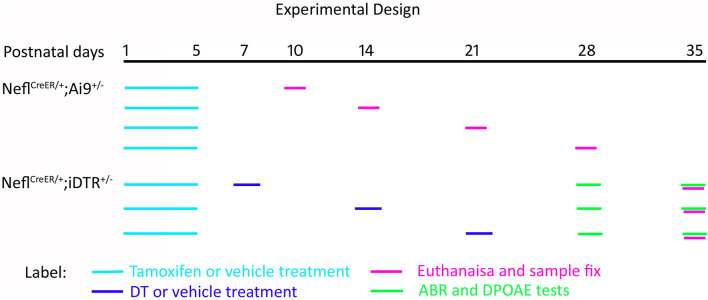
Schematic diagram of the experimental design. *Nefl*^*CreER/*+^;Ai9^+/–^ mice were treated with tamoxifen or vehicle on postnatal day 1 (P1) for 4–5 consecutive days. The pups were followed up and euthanized on P10, P14, P21, and P28. *Nefl*^*CreER/*+^;iDTR^+/–^ mice were treated with tamoxifen on P1–P5, followed by either vehicle or diphtheria toxin (DT) treatment on P7, P14, and P21. Hearing tests, including auditory brainstem response (ABR) and distortion product otoacoustic emission (DPOAE), were conducted on P28 and P35, and mice were euthanized on P35.

*Nefl*^*CreER/CreER*^ mice were crossed with the reporter strain Ai9 to obtain heterozygous *Nefl*^*CreER/*+^;Ai9^+/–^ offspring for the characterization of Cre activity in the cochlea. Tamoxifen or vehicle was administered to the dam *via* gavage on P1 for 4–5 days, and the pups received treatment *via* feeding. Tamoxifen (T5648, Sigma) was dissolved in corn oil (C8267, Sigma) at 10 mg/ml. Either tamoxifen (4 mg/40 g body weight) or corn oil was administered daily *via* oral gavage for 4–5 consecutive days (Koundakjian et al., 2007; Fang et al., 2012). Treated mice were followed up and euthanized on P10, P14, P21, and P28 for histology and immunofluorescence study to determine the Cre activity. *Nefl*^*CreER/CreER*^ mice were crossed with iDTR mice to obtain heterozygous *Nefl*^*CreER/*+^;iDTR^+/–^ mice in order to determine the SGN degeneration. For the DT treatment, *Nefl*^*CreER/*+^;iDTR^+/–^ mice were treated with tamoxifen as above, and a single dose of DT (List Biology Laboratories #150, 10 ng/g body weight, i.p.) was administered on P7, P14, or P21 (Figure 1).

### Hearing Tests

Animals received hearing tests at 4 and 5 weeks old prior to euthanasia. Distortion product otoacoustic emission (DPOAE) and ABR tests were used to study the function of outer hair cells and the auditory system using the RP2.1 and RZ6 systems [Tucker-Davis Technology (TDT), Alachua, FL, United States] (Hu et al., 2009; Zhang et al., 2011; Deng et al., 2019). The TDT System 3 software was applied for signal generation and auditory response collection. The ABR stimulation level ranged from 5 to 90 dB sound pressure level (SPL) in 5-dB steps using 8, 16, 24, and 32 kHz pure tone and click sound. The threshold was determined as the lowest stimulation decibel SPL that generated a wave II amplitude larger than 0.2 mV. At 16 and 24 kHz, the configuration of DPOAE was set as F2/F1 = 1.2 and L1 = L2 + 10 dB. L1 ranged from 10 to 80 dB SPL in 5-dB SPL steps. The DPOAE threshold was determined as the lowest level of DPOAE responses (dp) of at least 10 dB above the noise floor.

### Immunofluorescence and Imaging

Mice were anesthetized with CO_2_, followed by heart perfusion using saline (0.9% NaCl) and 4% paraformaldehyde (PFA; 158127, Sigma). The cochlear tissues were rapidly dissected and perfused with PFA. The dissected cochlear tissue was decalcified in 0.1 M EDTA for 1–2 weeks until the tissues softened, followed by surface preparation or cryosection at 10-μm thickness using our published methods (Hu et al., 2004; Zhang et al., 2011; Deng et al., 2019; Deng and Hu, 2020). Immunofluorescence was used for the detection of neuronal and hair cell proteins using our published methods (Hu et al., 2004; Zhang et al., 2011; Deng et al., 2019; Deng and Hu, 2020). The primary antibodies included anti-Nefl (1:200; sc-20012, Santa Cruz Biotechnology, Dallas, TX, United States), anti-beta III tubulin (TUJ1, 1:1,000; ab-2313564, Aves Labs, Tigard, OR, United States), and anti-myosin VIIa [1:200; 138-1-C, Developmental Studies Hybridoma Bank (DSHB), Iowa, City, IA, United States, and 25-6790, Proteus, Ramona, CA, United States]. Secondary antibodies were Alexa Fluor-488 (715-546-150), Cy3 (711-165-152), or Alexa Fluor-647 (703-606-155) conjugated antibodies (1:500; all from Jackson ImmunoResearch, West Grove, PA, United States). Leica SPE confocal microscope and DM2500 upright epifluorescence microscopes were used for observation and imaging.

### Quantitative Study and Statistical Analysis

In the quantitative study, the ABR and DPOAE data were analyzed using two-way analysis of variance (ANOVA) with *post-hoc* tests. The two factors were treatment type (DT and vehicle) and treatment time (P7, P14, and P21). *Post-hoc* tests were used to compare the vehicle and DT treatments in the P7, P14, and P21 groups. For cell counting, the cells and the area were calculated using the cell count and measurement modules of ImageJ software (NIH) using our published methods (Li et al., 2016; Hu et al., 2017, 2019, 2021; Liu et al., 2018). For SGN cell counting, the SGN area was determined, and *Nefl*-positive cells were calculated for the P7, P14, and P21 groups. The average cell number per 10^4^ μm^2^ was calculated for data analysis. All three cochlear turns were analyzed for the generation of data. For hair cell counting, surface preparation was performed to expose the hair cell epithelium, and myosin VIIa-positive cells were calculated at 100-μm distance for each animal using our published methods (Liu et al., 2018; Deng et al., 2019). Six animals were included in each group for statistical analysis of the ABR, DPOAE, and SGN cell counts, and five cochlear basilar membranes were dissected per group for analysis of the number of hair cells. A *p*-value of 0.05 was considered as the criterion of statistical significance.

## Results

### Characterization of the Cre Activity of *Nefl*^*CreER*/CreER^ Mouse Spiral Ganglion Neurons

**FIGURE 2:**
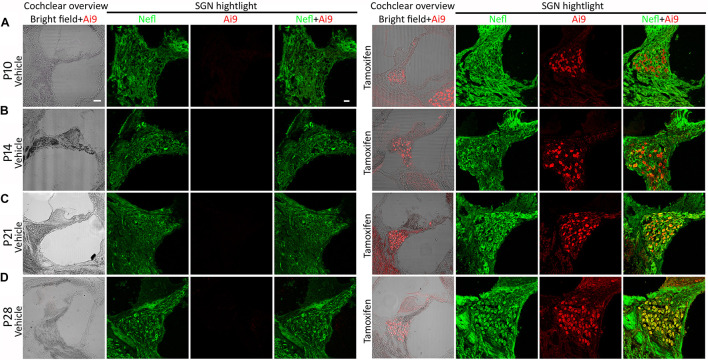
Cre activity of *Nefl*^*CreER/*+^ mice. *Nefl*^*CreER/CreER*^ mice were crossed with a reporter strain, Ai9, to obtain heterozygous *Nefl*^*CreER/*+^;Ai9^+/–^ mice, which were treated with tamoxifen or vehicle on postnatal day 1 (P1) for 4–5 consecutive days. The pups were euthanized on P10 **(A)**, P14 **(B)**, P21 **(C)**, and P28 **(D)**. In the vehicle group, no significant Ai9 tdTomato fluorescence was observed in the cochlear overview or spiral ganglion neuron (SGN) area highlight. However, robust Ai9 tdTomato fluorescence was identified in the cochlear overview and SGN area highlight in heterozygote mice treated with tamoxifen. In the SGN area, Ai9 tdTomato fluorescence was co-labeled with Nefl immunofluorescence in the tamoxifen groups, whereas only Nefl immunofluorescence was observed in the vehicle groups **(A–D)**, suggesting tamoxifen-induced Cre activity of *Nefl*^*CreER/*+^;Ai9^+/–^ mouse SGNs. *Scale*, 50 μm in cochlear overview and 20 μm in SGN highlight.

*Nefl* is expressed in developing and mature neurons, including SGNs (Sun et al., 2018). The *Nefl*^*CreER*/CreER^ mouse strain possessing a tamoxifen-inducible Cre cassette knocked into the *Nefl* gene was used in this study (Rotolo et al., 2008). To determine the *Nefl*-mediated CreER activity, the Cre reporter transgenic mouse strain Ai9 that has a LoxP-flanked STOP cassette preventing the transcription of a ubiquitous CAG promoter-driven tdTomato fluorescence was used. Tamoxifen or vehicle was administered to the dam *via* gavage on P1 for 4–5 days (Figure 1). The heterozygous *Nefl*^*CreER/*+^;Ai9^+/–^ pups received treatment *via* feeding and were followed up and euthanized on P10. It was observed that SGNs expressed both CreER-mediated tdTomato fluorescence and Nefl immunofluorescence, suggesting the CreER activity of *Nefl*^*CreER*/+^ at the neonatal stage (Figure 2A). It is known that SGNs develop during the postnatal period and mature around P21–28 (Shrestha et al., 2018; Sun et al., 2018), so we opted to determine the CreER activity during postnatal development. The dam was treated with tamoxifen or vehicle by gavage from P1 to P5 for 4–5 days, and the offspring received tamoxifen *via* feeding, followed by euthanasia on P14, P21, and P28. Robust tdTomato fluorescence was observed in SGNs from P14 to P28 in tamoxifen-treated *Nefl*^*CreER/*+^;Ai9^+/–^ offspring, which overlapped with the Nefl immunofluorescence (Figures 2B–D). In vehicle-treated *Nefl*^*CreER/*+^;Ai9^+/–^ offspring, only Nefl immunofluorescence was observed without tdTomato fluorescence (Figures 2B–D). This experiment suggests that *Nefl*^*CreER*/+^ mouse SGNs possess the inducible CreER activity during the postnatal development period.

### Functional Evaluation of the Spiral Ganglion Neuron Loss by Click and Pure Tone Auditory Brainstem Response

To generate an SGN damage model, the iDTR knock-in mouse strain was used. iDTR mice had the simian DTR insertion at the ROSA26 locus that is blocked by an upstream LoxP-flanked STOP sequence (Buch et al., 2005). When bred with Cre-recombinase-expressing *Nefl*^*CreER*/*CreER*^ mice, the STOP sequence was deleted in *Nefl*-Cre-expressing SGNs to allow DTR expression. Following DT treatment, iDTR-expressing SGNs were susceptible to ablation.

**FIGURE 3:**
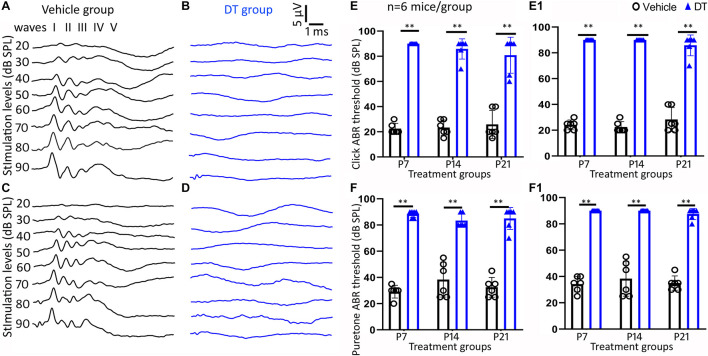
Auditory brainstem response (ABR) measurement study. Representative ABR waveforms of the vehicle and diphtheria toxin (DT) groups are shown in **(A–D)**. ABR was tested on 4- **(A,B)** and 5-week-old **(C,D)** *Nefl*^*CreER/*+^;iDTR^+/–^ mice that were treated with tamoxifen on P1–P5, followed by either vehicle **(A,C)** or DT **(B,D)** treatment. Quantitative analysis shows significant differences in click **(E,E1)** and pure tone (16 kHz) **(F,F1)** between the DT and vehicle groups in the P7, P14, and P21 treatment groups in the measurements at both 4 **(E,F)** and 5 weeks old **(E1,F1)**. ^∗∗^*p* < 0.01 (ANOVA, *n* = 6 mice per group).

Homozygous *Nefl*^*CreER/CreER*^ and iDTR mice were crossbred to obtain heterozygous *Nefl*^*CreER*/+^;iDTR^+/–^ offspring, which were treated with tamoxifen on P1–P5, followed by DT or vehicle injection on P7, P14, and P21 (Figure 1). Click and pure tone ABR tests were performed at 4 and 5 weeks old (Figure 3). It was found in the P7 treatment group that all DT-treated mice did not have an ABR response waveform following 90-dB SPL click stimulation (> 90 dB SPL) at 4 weeks old, whereas the threshold of vehicle-treated mice was 22.5 ± 4.2 dB SPL (mean ± SD) (Figure 3E). The click ABR threshold was similar a week later, at 5 weeks old: no response at 90 dB SPL in the DT group and remained normal (24.1 ± 3.8 dB SPL) in the vehicle group (Figure 3E1). In the P14 treatment group, the click ABR thresholds were 23.3 ± 6.1 and 85.8 ± 8.0 dB SPL for the vehicle and DT groups at 4 weeks old and were 22.5 ± 4.2 and > 90 dB SPL at 5 weeks old, respectively. In the P21 group, the click ABR thresholds for the vehicle and DT groups were 25.8 ± 11.1 and 76.3 ± 16.0 dB SPL at 4 weeks old and were 28.3 ± 9.3 and 83.8 ± 9.5 dB SPL at 5 weeks old, respectively (Figures 3E,E1). In the statistical analysis, two-way ANOVA and *post-hoc* tests were performed with two factors: treatment types (vehicle and DT) and treatment ages (P7, P14, and P21; *n* = 6 mice per group). In the test at 4 weeks old, the overall effects of treatment type and treatment age were not statistically significant [*F*_(__2, 30)_ = 1.595, *p* = 0.2196]. The effect of treatment age was also not statistically significant [*F*_(__2, 30)_ = 0.3451, *p* = 0.7109]. However, the effect of treatment type (vehicle vs. DT) was statistically significant [*F*_(__1, 30)_ = 459.7, *p* < 0.0001]. In the *post-hoc* test of the comparison of the vehicle and DT groups, significant differences were observed in the P7, P14, and P21 groups (*p* < 0.0001 for all three groups). In the test at 5 weeks old, the overall effects of treatment type and treatment age were not statistically significant [*F*_(__2, 30)_ = 2.831, *p* = 0.0748]. The effect of treatment age was also statistically insignificant [*F*_(__2, 30)_ = 0.09132, *p* = 0.9130]. However, the effect of treatment type was statistically significant [*F*_(__1, 30)_ = 1197, *p* < 0.0001]. In the *post-hoc* test of the comparison of the vehicle and DT groups, P7, P14, and P21 groups had significant differences (*p* < 0.0001 for all three groups).

In pure tone ABR, responses to the 16-kHz stimulation were analyzed (Figures 3F,F1). In the P7 group, the thresholds of the vehicle and DT groups were 29.2 ± 4.9 and 88.3 ± 2.6 dB SPL at 4 weeks old and were 34.2 ± 5.8 and > 90 dB SPL at 5 weeks old, respectively. For the P14 group, these thresholds became 32.5 ± 8.2 and 83.3 ± 5.2 dB SPL at 4 weeks old and 38.3 ± 13.3 and > 90 dB SPL at 5 weeks old, respectively. For the P21 group, the threshold values were 32.5 ± 7.6 and 82.5 ± 9.6 dB SPL at 4 weeks old and were 35.0 ± 5.5 and 86.3 ± 4.8 dB SPL at 5 weeks old, respectively. In the statistical analysis, two-way ANOVA and *post-hoc* tests were performed with the two factors: treatment type (vehicle and DT) and treatment age (P7, P14, and P21; *n* = 6 mice per group). In the test at 4 weeks old, the overall effects of treatment type and treatment age were not statistically significant [*F*_(__2, 30)_ = 2.500, *p* = 0.0990]. The effect of treatment age was also statistically insignificant [*F*_(__2, 30)_ = 0.2880, *p* = 0.7518]. However, the effect of treatment type (vehicle vs. DT) was statistically significant [*F*_(__1, 30)_ = 407.2, *p* < 0.0001]. In the *post-hoc* test of the vehicle and DT groups, significant differences were observed in the P7, P14, and P21 groups (*p* < 0.0001 for the three groups). In the test at 5 weeks old, the overall effects of treatment type and age were not statistically significant [*F*_(__2, 30)_ = 0.3387, *p* = 0.7154]. The effect of treatment age was statistically insignificant [*F*_(__2, 30)_ = 0.6290, *p* = 0.5400]. However, the effect of treatment type was statistically significant [*F*_(__1, 30)_ = 594.6, *p* < 0.0001]. In the *post-hoc* comparison of the vehicle and DT groups, the P7, P14, and P21 groups showed significant differences (*p* < 0.0001).

### Neuronal Protein Expression Changes

**FIGURE 4:**
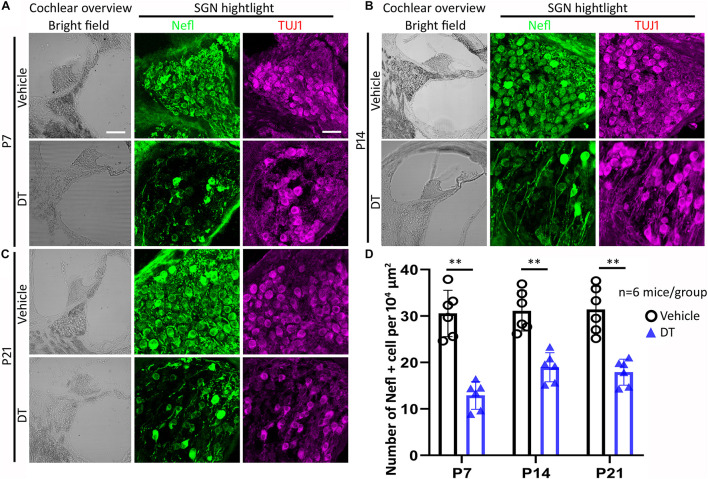
Spiral ganglion neuron (SGN) study of *Nefl*^*CreER/*+^;iDTR^+/–^ mice. *Nefl*^*CreER/*+^;iDTR^+/–^ mice were treated with tamoxifen, followed by diphtheria toxin (DT) or vehicle treatment on P7 **(A)**, P14 **(B)**, and P21 **(C)**. Nefl and TUJ1 immunofluorescence was used to identify SGNs. In the quantitative study **(D)**, significantly decreased numbers of Nefl-expressing SGNs were observed in the DT treatment groups, including the P7, P14, and P21 groups. ^∗∗^*p* < 0.01 (ANOVA, *n* = 6 mice per group). *Scale*, 100 μm in cochlear overview and 25 μm in SGN highlight.

Immunofluorescence using anti-Nefl and anti-TUJ1 antibodies was conducted to study the morphology and protein expressions of SGNs for the *Nefl*^*CreER/*+^;iDTR^+/–^ offspring at the end of the experiment. It was observed that SGNs expressed neuronal proteins Nefl and TUJ1 in the P7, P14, and P21 groups (Figures 4A–C). In the quantitative study, the number of Nefl-expressing cells was consistent in vehicle-treated groups, whereas it decreased in the P7, P14, and P21 groups treated with DT (Figure 4D). In vehicle-treated mice, the average numbers of Nefl-expressing cells per 10^4^ μm^2^ were 30.6 ± 5.0, 31.1 ± 4.3, and 31.4 ± 5.0 for the P7, P14, and P21 groups, respectively. In DT-treated groups, the average numbers of Nefl-expressing cells per 10^4^ μm^2^ were 12.9 ± 3.0, 19.0 ± 3.1, and 17.9 ± 2.8 for the P7, P14, and P21 groups, respectively. In the statistical analysis, two-way ANOVA and *post-hoc* tests were performed with two factors: treatment type (vehicle and DT) and treatment age (P7, P14, and P21; *n* = 6 mice per group). The overall and the treatment age effects were not statistically significant [*F*_(__2, 30)_ = 3.065, *p* = 0.0916, and *F*_(__2, 30)_ = 1.677, *p* = 0.2355, respectively]. However, the effect of treatment type (vehicle vs. DT) was statistically significant [*F*_(__1, 30)_ = 80.64, *p* = 0.0003]. In the *post-hoc* comparison of the vehicle and DT groups, significant differences were observed (*p* = 0.0002, 0.0042, and 0.0018 for the P7, P14, and P21 groups, respectively) (Figure 4).

### Hair Cell Function and Protein Expression Study

**FIGURE 5:**
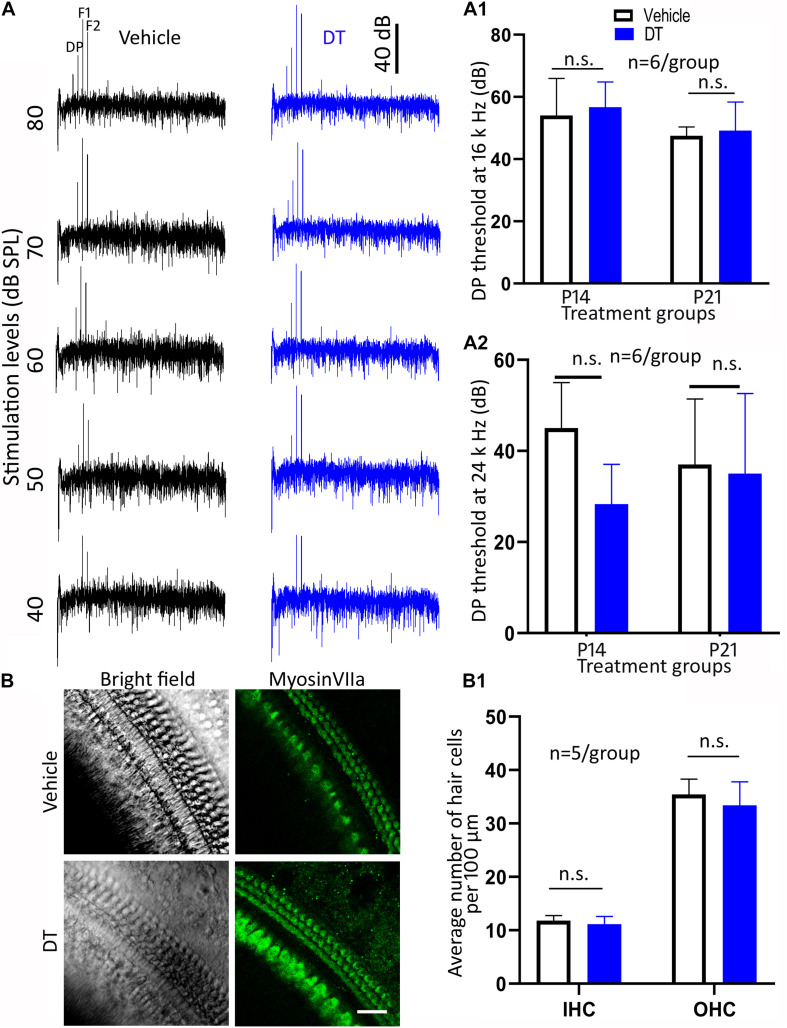
Hair cell protein expression and distortion product otoacoustic emission (DPOAE) study. Representative DPOAE waveforms of *Nefl*^*CreER/*+^;iDTR^+/–^ mice treated with either diphtheria toxin (DT) or vehicle are shown in **(A)**. In the quantitative study, DPOAE measurements at 4 weeks old did not show a significant difference between the DT and vehicle treatments at 16 **(A1)** or 24 kHz **(A2)** in the P14 or P21 groups (two-way ANOVA: *p* > 0.05, *n* = 6 mice/group). In the surface preparation of *Nefl*^*CreER/*+^;iDTR^+/–^ mice treated with either DT or vehicle on P14, the numbers of myosin VIIa-expressing cells are statistically insignificant (**B,B1**; ANOVA: *p* > 0.05, *n* = 5 mice/group). *n.s.* statistically insignificant. *Scale*, 25 μm in **(B)**.

To evaluate the hair cell function of the *Nefl*^*CreER/*+^;iDTR^+/–^ offspring, DPOAE was performed. The DPOAE thresholds were determined in the P14 and P21 groups at 4 weeks old (Figure 5A). In the statistical analysis using two-way ANOVA, the overall and individual effects of treatment type (vehicle vs. DT) and treatment age (P14 vs. P21; *n* = 6 mice per group) at 16-kHz stimulation were not statistically significant (*p* > 0.05): *p* = 0.9108, 0.6062, and 0.1360 for the overall, treatment type, and treatment age effects, respectively (Figure 5A1). In the analysis of the 24-kHz stimulation, these numbers became *p* = 0.2640, 0.1601, and 0.9175, respectively (Figure 5A2). These data suggest that the DPOAE thresholds were not significantly different between the DT and vehicle groups.

In the hair cell protein expression study, anti-myosin VIIa antibodies were used to identify hair cells using the basilar membrane surface preparation for the P14 group (Figure 5B). In the quantification study, the average numbers of inner hair cells were 11.9 ± 0.96 and 11.1 ± 1.5 per 100 μm for the vehicle and DT groups, whereas the numbers for outer hair cells were 35.4 ± 2.87 and 33.4 ± 4.42, respectively (Figure 5B1). Statistical analysis showed no significant difference between the vehicle and DT groups (ANOVA, *n* = 5 mice per group): the *p*-values were 0.9138 and 0.4626 for inner and outer hair cells, respectively.

These data show that hair cell number and function were not statistically different between the DT and vehicle groups, suggesting no hair cell damage was observed in this animal model.

## Discussion

In this study, it was found that *Nefl*^*CreER*/CreER^ mice exhibited Cre activity during the postnatal period from P1 to P28. When bred with iDTR mouse, the offspring *Nefl*^*CreER/*+^;iDTR^+/–^ were responsive to DT treatment and demonstrated damage specific to SGNs in the cochlea on P7–P21, which was indicated by the functional ABR test and SGN cell counts. DPOAE and cell counting suggest that hair cells were not affected following DT treatment. These data indicate that the SGNs of *Nefl*^*CreER/*+^;iDTR^+/–^ mice could be specifically damaged by DT treatment in the cochlea.

*Nefl* encodes Nefl, which is expressed in developing and mature neurons, including SGNs (Torkos et al., 2008; Sun et al., 2018). A previous study has shown that the *Nefl*^*CreER*/+^ mouse strain shows neuronal-specific Cre activity in the central nervous system (Rotolo et al., 2008). However, the auditory system Cre activity of this mouse strain has not been determined. In this study, *Nefl*^*CreER/CreER*^ mice were crossed with a reporter mouse strain, the Ai9 mouse, to obtain *Nefl*^*CreER/*+^;Ai9^+/–^ offspring in order to determine the Cre activity in the auditory system. Tamoxifen was administered to the offspring *via* feeding from dams that had been gavaged with tamoxifen. It was found that tamoxifen-treated *Nefl*^*CreER/*+^;Ai9^+/–^ pups showed robust Ai9 tdTomato fluorescence in the SGN area and the nerve projections, whereas vehicle-treated *Nefl*^*CreER/*+^;Ai9^+/–^ pups did not show Ai9 fluorescence. The Ai9 fluorescence totally overlapped with Nefl immunofluorescence. Additionally, tamoxifen-treated mice showed Cre activity during the postnatal period, from P1 to P28. These data are consistent with previous reports of Cre activity in central nervous system neurons (Rotolo et al., 2008). Importantly, the Cre activity is robustly inducible for at least 28 days after birth, which is useful for the generation of a postnatal and young adult SGN damage model using the iDTR transgenic mouse model.

It is known that the hearing onset of mouse is around postnatal days 12–14 (Ehret, 1976; Kamiya et al., 2001; Romand, 2003) and SGN development and subtype characterization progress during the postnatal period up to P28 (Shrestha et al., 2018; Sun et al., 2018). A previous study has reported on an SGN damage model following DT treatment on P21 (Pan et al., 2017). Whether SGNs respond to damage in the pre-hearing and hearing onset periods remains unclear. To generate a specific SGN damage model, in the present study, homozygous *Nefl*^*CreER/CreER*^ mice were crossed with iDTR mice, and the heterozygous offspring were exposed to either DT or vehicle on P7, P14, and P21. Nefl and TUJ1 immunostaining was used to evaluate the expressions of the neuronal proteins of SGNs, and Nefl-expressing cells were used to quantitatively examine the number of surviving SGNs. It was found that approximately 58, 39, and 43% SGNs were damaged in the P7, P14, and P21 groups, respectively. In a previous study, roughly 30–40% of SGN loss was observed 7 days post-DT injection in the SGN-damaged Cre-positive group (Pan et al., 2017). In the present study, the SGN loss at 2–3 weeks post-DT treatment in the P14 and P21 groups was similar to that in the previous study using a different Cre mouse strain, the *Bhlhb5*^*CreER/*+^ mouse strain. However, the SGN loss at 4 weeks post-DT treatment in the P7 group was approximately 58%, which was significantly larger than that in the P14 and P21 groups. The difference may be related to the treatment time, and the pre-hearing damage to the SGN on P7 may have caused more severe neuronal degeneration than did post-hearing insults. The follow-up time post-DT treatment may have also contributed to the different damage levels, which may require additional experiments in our future studies. These data suggest that DT treatment on P7 at the pre-hearing stage may cause a more significant SGN loss.

In the functional assays, click and pure tone ABR tests were performed to evaluate the function of the auditory system at 4 and 5 weeks old. Compared to vehicle-treated groups, significant click ABR threshold shifts (>50–60 dB SPL) were observed in DT-treated animals in the P7, P14, and P21 groups. In the pure tone ABR test at 16 kHz, the threshold shifts between the DT- and vehicle-treated animals were around 50–55 dB SPL in the P7, P14, and P21 groups. These results were different from those of a previous study, in which the ABR thresholds largely overlapped in the presence and absence of Cre activity following DT treatment using *Bhlhb5*^*CreER/*+^;iDTR mice. The reason for this discrepancy is unclear. One possibility might be attributed to the neuronal gene that mediates the Cre activity. The *Bhlhb5* gene was used to mediate Cre activity in the previous report, whereas the *Nefl* gene was selected for the current study. *Nefl* is robustly expressed along the nerve projections of bipolar SGNs, and Cre-mediated iDTR-positive nerve projections may have been significantly damaged in response to DT treatment, which may have caused SGN–hair cell disconnection and subsequent hearing threshold changes. Further research may be required to understand the ABR threshold shifts in this mouse strain. In the meantime, these functional data show that significant hearing function changes can be achieved following DT treatment using the *Nefl*^*CreER/CreER*^ mouse strain, which may serve as a useful animal model for SGN degeneration study.

Hair cell protein expression and DPOAE tests were used to evaluate whether hair cells are affected in the *Nefl*^*CreER/*+^;iDTR^+/–^ mouse strain in this study. It was found that the DPOAE thresholds were statistically insignificant between the DT and vehicle groups, suggesting that the outer hair cell function is not compromised following DT treatment. In hair cell protein expression and hair cell counting using basilar membrane surface preparation, both inner and outer hair cells expressed the hair cell protein myosin VIIa in the DT and vehicle groups. In cell counting, no significant difference was identified between the vehicle and DT groups, suggesting that hair cell loss was not observed. These functional, protein expression, and morphology data suggest that hair cells were not affected post-DT treatment in the *Nefl*^*CreER/*+^;iDTR^+/–^ mouse model.

In summary, inducible *Nefl*-CreER-mediated Cre activity was identified in Nefl^*CreER/*+^;Ai9^+/–^ mouse SGNs for at least 28 days after birth. When crossed with an iDTR mouse strain, the offspring *Nefl*^*CreER/*+^;iDTR^+/–^ showed SGN loss following a single dose of DT injection. Compared to vehicle-treated mice, both SGN number and ABR thresholds were significantly changed in DT-injected *Nefl*^*CreER/*+^;iDTR^+/–^ mice. The inner and outer hair cell numbers and the DPOAE thresholds were not significantly changed between the DT- and vehicle-treated groups. These data suggest that DT treatment can specifically target SGNs of the postnatal *Nefl*^*CreER/*+^;iDTR^+/–^ mouse model in the cochlea.

There are some limitations to this SGN damage model. The present study focused on SGN evaluation, whereas damage to other types of neurons, such as the central auditory neurons, has not been determined. Additionally, this report focused on SGN damage assays before, around, and after hearing onset, while SGN damage of mature mice has not been identified. These limitations should be addressed in future independent experiments. Taken together, this report identified a mouse model with inducible damage specific to the neuronal lineage in the cochlea, which can be used to further characterize primary SGN degeneration without interfering with hair cells. The mouse model reported in this study may prove to be a powerful mammalian model to investigate the development of postnatal SGNs, degeneration of SGNs, prevention of SGN damage, and regeneration of SGNs, which may provide insights into SGN research in the future.

## Data Availability Statement

The original contributions presented in the study are included in the article/supplementary material, further inquiries can be directed to the corresponding author/s.

## Ethics Statement

The animal study was reviewed and approved by the Institutional Animal Care and Use Committee (IACUC) at Wayne State University.

## Author Contributions

ZH designed the project. ZH, FK, AS, and MD performed the experiment, analyzed the data, and wrote the manuscript. All authors contributed to the article and approved the submitted version.

## Conflict of Interest

The authors declare that the research was conducted in the absence of any commercial or financial relationships that could be construed as a potential conflict of interest.

## Publisher’s Note

All claims expressed in this article are solely those of the authors and do not necessarily represent those of their affiliated organizations, or those of the publisher, the editors and the reviewers. Any product that may be evaluated in this article, or claim that may be made by its manufacturer, is not guaranteed or endorsed by the publisher.
